# The disparities of healthcare access for adults with autism spectrum disorder: Protocol for a systematic review

**DOI:** 10.1097/MD.0000000000014480

**Published:** 2019-02-15

**Authors:** Shenae Calleja, Fakir M. Amirul Islam, Jonathan Kingsley, Rachael McDonald

**Affiliations:** a Faculty of Health, Arts and Design, Swinburne University of Technology, Hawthorn; b School of Population and Global Health, University of Melbourne, Carlton, Victoria, Australia.

**Keywords:** adults, autism spectrum disorder, barriers, enablers, healthcare access

## Abstract

**Introduction:**

Adults living with autism spectrum disorder (ASD) can experience many factors that may impact their everyday lives. Striving for optimal health and enduring a healthy lifestyle comes with the ability to access appropriate healthcare services, yet adults with ASD have unmet healthcare needs. The barriers and enablers of healthcare access for adults with ASD remain unclear. We will conduct a systematic review to explore what is currently known about healthcare access for adults with ASD, this will determine the level and appropriateness of access to healthcare services to better support the lives of adults with ASD.

**Method and analysis:**

The systematic review will report on all studies that include quantitative, qualitative, and mixed-methods designs that consider healthcare access for adults with ASD. We will search 5 databases: EBSCOhost, Scopus, PubMed, the Cochrane Library, and Web of Science. The Mixed Methods Appraisal Tool (MMAT) will be used to assess quality of articles and the Cochrane RoB 2.0 Tool will be used to assess for bias. Clarifying the evidence in this area will be important for future research directions when developing and piloting health interventions for researchers and healthcare clinicians in the field.

**Ethics and dissemination:**

There are no human participants, data, or tissue being directly studied for the purposes of the review; therefore, ethics approval and consent to participate is not applicable.

**Registration and Status:**

PROSPERO 2018 CRD42018116093.

## 1 Introduction

Autism spectrum disorder (ASD) is a neurodevelopmental disorder and is characterized by impaired social communication and social interaction skills and people on the autism spectrum may experience restricted interests and repetitive behaviours.^[1–4]^ ASD is typically diagnosed using the criteria from the Diagnostic and Statistical Manual of Mental Disorders, 5th edition: DSM-5.^[3]^ It is estimated that globally 1 in 160 people have a diagnosis of ASD.^[5]^ In Australia, it is estimated that 164,000 people have a diagnosis of ASD, which affects approximately 1 in 150 people^[6]^ and therefore the research team viewed this as a critical research area. Further, ASD is mainly diagnosed during the childhood developmental stage around the age of 4 to 5^[7]^ and is diagnosed more commonly in males than females, with a 4:1 male-to-female ratio.^[7–10]^

This review focuses on access to healthcare for adults with ASD. While access to high-quality healthcare for individuals with developmental disabilities has been a public health concern since the deinstitutionalization of this population in the 1970s,^[11]^ people with ASD, still experience difficulties with accessing appropriate healthcare. This can be due to multiple factors, including the diagnosis itself, what age they were diagnosed, access and delivery of appropriate healthcare services, their living situation, family and friend support, level of education, and employment opportunities.^[12,13]^ People with ASD are frequently reported to demonstrate behaviors of concern such as aggression, property destruction, disruptive, and self-injurious behavior.^[10,14–16]^

A study conducted in the United States examined health conditions and functional status in adults with ASD and compared the results with the general population.^[17]^ Most notably, the researchers found a higher prevalence of seizure disorders, hypertension and allergies and lower rates of migraine headaches, sexually transmitted infections, tobacco use, and alcohol misuse, which is consistent with prior literature.^[4]^ Other studies indicate that many common chronic health conditions were significantly more common in adults with ASD.^[17,18]^

People with ASD are more likely to experience mental health conditions as their peers,^[19]^ with anxiety, bipolar disorder, dementia, depression, and schizophrenic disorder are common mental health conditions which are frequently observed among adults with ASD and are a particular area of concern.^[4,17,20–22]^ Comorbid psychiatric disorders may be more prevalent due to social challenges, challenges accessing health services, and appropriate interventions.^[23]^ Psychiatric disorders may go unrecognized and therefore may be at a heightened risk of developing mental health conditions.^[23]^

Striving for optimal health and enduring a healthy lifestyle comes with the ability to access appropriate healthcare services, yet adults with ASD have unmet healthcare needs^[24]^ which may impact their everyday lives and ultimately affect other factors of their lives such as their living situation, employment, and educational opportunities.^[25]^ While there are several reviews on disparities in healthcare for paediatrics, barriers for vaccinations, and autism intervention,^[26–29]^ none has been conducted in a systematic approach to report on all the published literature focusing on adults accessing healthcare services. Tregnago and Cheak-Zamora^[30]^ review considered disparities in healthcare, but the selected studies were based on whether differences exist for children with ASD compared with children without ASD and only in the United States. No reviews have considered the healthcare access for adults with ASD and the level of healthcare where barriers and enablers are present. For the first time, we are seeing a substantial number of people living with ASD moving into adulthood and using adult services. The healthcare access for adults with ASD is important, and a review that aims to identify known barriers and enablers internationally may assist when implementing appropriate health interventions in the future.

The healthcare services for other countries are diverse although, if they have developed an appropriate tool/resource to advance healthcare access for adults with ASD, this could undoubtedly be piloted, implemented, and evaluated in Australia. A systematic review of the literature to explore what evidence for access or otherwise to appropriate healthcare for adults with ASD is needed to determine the level of healthcare that may need further support, to better the lives of adults with ASD accessing appropriate healthcare services and for future research directions when developing and piloting health interventions. Completing an up-to-date systematic review is important and relevant for clinical practice and evidence for future intervention development.^[31]^

## 2 Objectives

The overall objective is to undertake a systematic review to answer the questions:

1. what are the barriers and enablers of healthcare access for adults with ASD, and
2. how can healthcare access for adults living with ASD be enhanced?

## 3 Method

This review is registered on the PROSPERO database CRD42018116093. Reporting will follow the Preferred Reporting Items for Systematic Reviews and Meta-Analyses (PRISMA) guidelines.

## 4 Eligibility criteria of inclusion of studies

### 4.1 Types of studies

This systematic review is mixed-methods and will identify all quantitative, qualitative, and mixed-methods research studies that consider healthcare access for adults with ASD. There is a paucity of high-quality quantitative studies in this area; thus, all quantitative methodologies have been included.^[9,32]^ Mixed method and qualitative studies have been included as scans of the literature indicate that there are a large number of this type of study and they give a level of detail and richness that is not present in the quantitative studies.^[33]^

#### 4.1.1 Inclusion criteria

For the purpose of this review, the study will include a primary diagnosis of ASD although ID is a cooccurring condition^[17]^ and will be included as a cocondition. The study types will be original peer-review research articles with a date from 2003-present—a 16-year range. The participants for our review are considered to be adults (over 18 years of age) with a primary diagnosis of ASD.

#### 4.1.2 Exclusion criteria

The search is limited to English and the search will be limited to adults living with ASD. No unpublished data will be included. Our exclusion criteria are pediatric studies—children under the age of 18, ASD not the primary diagnosis, studies of parents of children under the age of 18, review papers (systematic or narrative), book chapters, commentary articles, opinions, letters, and editorials.

### 4.2 Outcomes

The primary outcome will be to identify barriers and enablers of healthcare access for adults with ASD. From the outcomes identified, the level of healthcare that needs further support to better healthcare service access will be determined.

### 4.3 Information sources and search method

Five databases will be searched: EBSCOhost, Scopus, PubMed, The Cochrane Library and Web of Science, and the journal *Autism* will be hand searched. The search strategy was developed to capture all studies that would meet the above eligibility criteria and utilize appropriate journals. The database search strategies were developed with the assistance of a university librarian with experience in systematic reviews. The full search strategy is included (Table 1).

**Table 1:**
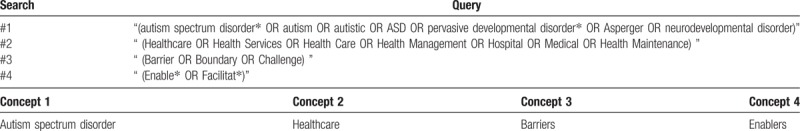
Key terms for database search full search strategy.

An example of a search strategy used in multiple databases to extract relevant articles is (Autism Spectrum Disorder OR Autism OR ASD OR Neurodevelopmental Disorder OR Asperger's OR Pervasive Developmental Disorder) AND (Healthcare OR Health Services OR Health Care OR Health Management OR Hospital OR Medical OR Health Maintenance) AND (Barrier OR Boundary OR Challenge) AND (Enable∗ OR Facilitat∗)).

### 4.4 Identification and selection of studies

The titles and abstracts of all studies generated through the combined database searches will be merged using Endnote X8^[34]^ a reference management software and the duplicates will be removed. One author (SC) will independently screen search results against eligibility criteria. All studies that meet the eligibility criteria on screening titles and abstracts will be sourced and read in full. One other author will review and screen the titles and the results and compare with the eligibility criteria, to increase validity (RM, AI, JK).^[35]^ We will resolve any disagreements though discussion (SC, RM, AI, JK). The search strategy and study selection processes will be documented using a PRISMA study flow diagram.^[36]^

### 4.5 Data extraction

One author (SC) will independently extract data from selected studies on key components addressing our research questions. A standardized data extraction form will be used to extract data from the included studies for assessment of quality and evidence synthesis (Table 2). Extracted information will include;

**Table 2:**
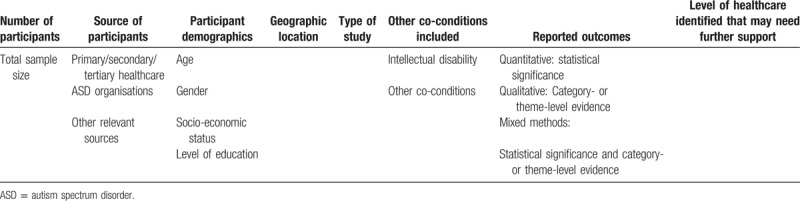
Sample elements for data extraction.

Quantitative studies: number of participants; source of participants (primary healthcare, secondary healthcare, tertiary healthcare); participant demographics (age, gender); geographic location; type of study; other coconditions included and reported outcomes and statistical significance; level of healthcare identified that may need further support.

Qualitative studies: number of participants; source of participants (primary healthcare, secondary healthcare, tertiary healthcare); participant demographics (age, gender); geographic location; type of study; other coconditions included and category- or theme-level evidence from the findings or results section of the included papers; level of healthcare identified that may need further support.

Mixed method studies: number of participants; source of participants (primary healthcare, secondary healthcare, tertiary healthcare); participant demographics (age, gender); geographic location; type of study; other coconditions included and reported outcomes and evidence from the findings or results section of the included papers; level of healthcare identified that may need further support.

In accordance with the current DSM-5 recommendations, studies that have classified their study participants as “autistic disorder,” “Asperger disorder,” or “pervasive developmental disorder not otherwise specified” will be given the diagnosis of ASD. Of importance is the level of healthcare that study authors have sourced participants and have investigated. We intend to report on the level of healthcare that may experience the greatest healthcare access barriers and enablers for adults with ASD.

### 4.6 Assessing of study quality

The methodology for this review was developed using the PRISMA-P protocol^[36]^ (checklist attached in additional file 3). The Mixed Methods Appraisal Tool (MMAT) will be used to assess evidence quality for the quantitative, qualitative, and mixed method studies.^[37,38]^ The MMAT permits us to appraise and describe methodological quality for the 3 domains.^[37,38]^ Risk of bias will be assessed using the Cochrane RoB 2.0 Tool to assess the potential biases of studies regarding study design and other factors.^[39]^ One author (SC) will appraise all the evidence. A randomized selection of 10 articles will be then also appraised by the coauthors and assessed for agreement using Cohen Kappa statistic. Where there are disagreements, these will be discussed and agreed upon. A further 10 random articles will then be scored by all authors and assessed for inter-rater reliability. Recommendations will be provided for practice and policy-making if sufficient high-quality evidence exists, or future directions will be recommended for research to fill existing gaps in knowledge or to strengthen the body of evidence.

### 4.7 Data synthesis

Search results will be summarized in a PRISMA Protocol^[35]^ First, the studies will be categorized according to study design and then the study characteristics.

For quantitative studies: Frequencies and percentages will be reported for categorical variables and the means and standard deviations for continuous variables, depending on the data. Where appropriate, pooling of data and meta-analysis will be performed.

For qualitative studies: We anticipate the qualitative data will describe the perspectives of patients with ASD and their primary carers accessing healthcare services and the barriers and enablers they may identify. The qualitative data will be analyzed using the MMAT, identifying patterns (themes) within the data. Researchers will determine categories based on the analysis.

Where there is unreported data and/or clarification is required to determine if the study can be included, we (SC) will attempt to contact the study authors, to obtain the missing data using a maximum of 3 emails. If data cannot be obtained, we will analyze the available data and in the discussion section, report the potential impact.

## 5 Discussion

This review aims to report the known healthcare access barriers and enablers faced by adults with ASD. The study will use quantitative, qualitative, and mixed methods designs. First, it is imperative to identify and understand the different barriers and enablers people with ASD experience as ASD is a complex lifelong condition, which can have a potentially detrimental impact on adult functioning. Second, it assists in identifying pathways for future pilot interventions that contribute to healthcare for adults with ASD in providing opportunities to better improve healthcare access by developing appropriate resources/tools. Finally, clarifying the current literature focusing on adults with ASD and healthcare access is important for streamlining and directing further research efforts for future interventions.

## 6 Ethics and dissemination

There are no human participants, data, or tissue being directly studied for the purposes of the review; therefore, ethics approval and consent to participate is not applicable. The results of this systematic review will be presented at international conferences and published in peer-reviewed journals.

## Author contributions

SC wrote the full first draft of the protocol. All other authors contributed to the editing process prior to submission. All authors read and approved the final manuscript.

**Supervision:** Rachael McDonald, Fakir M Amirul Islam, Jonathan Kingsley.

**Writing – original draft:** Shenae Calleja.

**Writing – review & editing:** Shenae Calleja, Rachael McDonald, Fakir M Amirul Islam, Jonathan Kingsley.
